# Supplementary material for: The complex relationship of exposure to new Plasmodium infections and incidence of clinical malaria in Papua New Guinea
Source: eLife. 2017 Sep 1;6:e23708. doi: 10.7554/eLife.23708 (PMC5606846; doi:10.7554/eLife.23708)
Supplement: Supplementary file 1. — Table 1. Univariate/PQ-adjusted predictors for time to recurrent blood-stage infection with Plasmodium species by qPCR. Table 2. Univariate (P. vivax) and univariate/PQ-treatment-adjusted (P. falciparum) predictors for Pv- and Pf-molFOB by follow-up interval. Table 3. Univariate/PQ-treatment-adjusted predictors for time to P. vivax and P. falciparum episodes. Table 4. Univariate/PQ-treatment-adjusted predictors for odds of P. falciparum clinical episodes. [file elife-23708-supp1.docx]

**Supplementary File 1 – Univariate factors**

**Table 1. Univariate/PQ-adjusted predictors for time to recurrent blood-stage infection with *Plasmodium* species by qPCR.**

| **Variable** | ***P. vivax*** | | | ***P. falciparum*** | | | ***P. malariae*** | | | ***P. ovale*** | | |
| --- | --- | --- | --- | --- | --- | --- | --- | --- | --- | --- | --- | --- |
|  | **HR^1^** | **CI_95_** | ***p*-value** | **HR^1^** | **CI_95_** | ***p*-value** | **HR^1^** | **CI_95_** | ***p*-value** | **HR^1^** | **CI_95_** | ***p*-value** |
| PQ treatment | 0.27 | 0.20-0.36 | <0.001 | 0.79 | 0.57-1.09 | 0.057 | 0.60 | 0.26-1.38 | 0.233 | 0.40 | 0.17-0.96 | 0.040 |
| Age | 0.94 | 0.86-1.02 | 0.155 | 0.97 | 0.87-1.08 | 0.542 | 0.95 | 0.73-1.23 | 0.684 | 0.90 | 0.69-1.18 | 0.447 |
| LLIN use at enrolment | 0.38 | 0.25-0.58 | <0.001 | 0.44 | 0.27-0.72 | 0.001 | 0.77 | 0.18-3.28 | 0.724 | 0.51 | 0.15-1.70 | 0.271 |
| Hb at enrolment (g/dl) | 0.81 | 0.73-0.89 | <0.001 | 0.76 | 0.68-0.86 | <0.001 | 0.66 | 0.50-0.87 | 0.003 | 0.78 | 0.58-1.04 | 0.087 |
| Village |  |  |  |  |  |  |  |  |  |  |  |  |
| Albinama (ref) | 1 |  |  | 1 |  |  | 1 |  |  | 1 |  |  |
| Amahup | 0.44 | 0.28-0.69 | <0.001 | 0.55 | 0.29-1.05 | 0.068 | 0.32 | 0.06-1.67 | 0.178 | 2.57 | 0.27-24.73 | 0.414 |
| Balanga | 2.23 | 1.46-3.39 | <0.001 | 2.06 | 1.15-3.69 | 0.016 | 1.34 | 0.35-5.12 | 0.665 | 7.28 | 0.81-65.11 | 0.076 |
| Balif | 0.96 | 0.63-1.50 | 0.843 | 0.57 | 0.29-1.12 | 0.101 | 0.21 | 0.02-1.79 | 0.153 | 4.13 | 0.46-36.92 | 0.205 |
| Bolumita | 4.92 | 3.29-7.36 | <0.001 | 6.79 | 4.15-11.12 | <0.001 | 3.08 | 1.04-9.11 | 0.041 | 22.40 | 2.88-174.39 | 0.003 |
| Numangu | 0.91 | 0.48-1.71 | 0.765 | 2.43 | 1.25-4.73 | 0.009 | 1.36 | 0.26-7.04 | 0.711 | 3.60 | 0.22-57.66 | 0.365 |
| Infection status at enrolment (by qPCR) | | | | | | | | | | | | |
| Uninfected (ref) | 1 |  |  | 1 |  |  | 1 |  |  | 1 |  |  |
| *P. vivax* | 1.34 | 0.97-1.87 | 0.079 | 1.49 | 0.94-2.37 | 0.090 | 1.03 | 0.23-4.62 | 0.971 | 1.97 | 0.63-6.23 | 0.246 |
| *P. falciparum* | 2.52 | 1.64-3.89 | <0.001 | 3.11 | 1.77-5.46 | <0.001 | 6.09 | 1.63-22.82 | 0.007 | 2.89 | 0.69-12.12 | 0.146 |
| *P. malariae* | 1.37 | 0.63-2.98 | 0.426 | 1.74 | 0.68-4.45 | 0.250 | 9.81 | 2.17-44.38 | 0.003 | 2.63 | 0.31-22.63 | 0.378 |
| Mixed *P.f. or P.v*.^2^ | 3.39 | 2.37-4.87 | <0.001 | 5.84 | 3.75-9.10 | <0.001 | 6.64 | 2.03-21.72 | 0.002 | 5.50 | 1.79-16.88 | 0.003 |

^1^ PQ treatment, univariate HR. All other HRs are adjusted only for PQ treatment. HRs were modeled using Cox proportional hazard regression.

^2^ Mixed infection including *P. falciparum* or *P. vivax* infection in conjunction with one or more other *Plasmodium spp.*

**Table 2. Univariate (*P. vivax*) and univariate/PQ-treatment-adjusted (*P. falciparum*) predictors for *Pv-* and *Pf-*_mol_FOB by follow-up interval.**

| **Variable** | ***P. vivax*** | | | | | | ***P. falciparum*** | | |
| --- | --- | --- | --- | --- | --- | --- | --- | --- | --- |
|  | **PQ arm** | | | **placebo arm** | | | **Combined arms** | | |
|  | **IRR^1^** | **CI_95_** | ***p*-value** | **IRR^1^** | **CI_95_** | ***p*-value** | **IRR^2^** | **CI_95_** | ***p*-value** |
| PQ treatment | n.a.^3^ | n.a. | n.a | n.a. | n.a. | n.a. | 0.94 | 0.64-1.39 | 0.785 |
| New *P. falc.* infections in interval | 1.78 | 1.18-2.69 | 0.006 | 1.52 | 1.11-2.10 | 0.010 | n.a. | n.a | n.a |
| New *P. vivax* infections in interval | n.a. | n.a | n.a. | n.a. | n.a. | n.a. | 1.41 | 1.13-1.75 | 0.002 |
| Age | 0.79 | 0.67-0.93 | 0.004 | 0.94 | 0.85-1.04 | 0.23 | 0.94 | 0.82-1.18 | 0.39 |
| LLIN at enrol. | 0.58 | 0.32-1.02 | 0.060 | 0.50 | 0.34-0.74 | <0.001 | 0.53 | 0.31-0.91 | 0.022 |
| Haemoglobin at enrol. (g/dl) | 0.90 | 0.79-1.01 | 0.081 | 0.94 | 0.86-1.03 | 0.20 | 0.84 | 0.77-0.91 | <0.001 |
| Village |  |  |  |  |  |  |  |  |  |
| Albinama (ref) | - | - |  | - | - |  | - | - |  |
| Amahup | 0.02 | 0.00-0.12 | <0.001 | 0.50 | 0.30-0.83 | 0.007 | 0.52 | 0.2-1.06 | 0.072 |
| Balif | 0.93 | 0.41-2.11 | 0.874 | 1.66 | 1.09-2.52 | 0.019 | 1.96 | 1.06-3.64 | 0.032 |
| Balanga | 0.25 | 0.09-0.74 | 0.013 | 1.00 | 0.64-1.56 | 1.000 | 0.74 | 0.36-1.52 | 0.416 |
| Bolumita | 1.98 | 1.05-3.78 | 0.036 | 3.18 | 2.19-4.59 | <0.001 | 7.80 | 4.48-13.55 | <0.001 |
| Numangu | 0.46 | 0.14-1.54 | 0.213 | 0.75 | 0.40-1.44 | 0.391 | 2.85 | 1.45-5.62 | 0.002 |
| Study Day |  |  |  |  |  |  |  |  |  |
| Day 0-35 (ref) | - | - |  |  |  |  | - | - |  |
| Day 36-80 | 1.46 | 0.60-3.55 | 0.410 | 1.90 | 1.21-2.75 | 0.001 | 2.54 | 1.45-4.45 | 0.001 |
| Day 81-175 | 1.35 | 0.61-2.99 | 0.450 | 0.81 | 0.5-1.19 | 0.285 | 1.12 | 0.67-1.88 | 0.655 |
| Day >175 | 0.66 | 0.26-1.67 | 0.480 | 0.56 | 0.37-0.84 | 0.005 | 0.91 | 0.49-1.71 | 0.772 |

^1^ *P. vivax*, stratified by treatment arm. Univariate IRRs were modeled per sampling interval using negative binomial generalized estimating equations allowing for repeated visits with log-link and an exchangeable correlation structure.

^2^ *P. falciparum*, combined treatment arms. PQ treatment, univariate IRR. All other IRRs are adjusted only for PQ treatment. IRRs were modeled per sampling interval using negative binomial generalized estimating equations allowing for repeated visits with log-link and an exchangeable correlation structure.

^3^ n.a., not applicable.

**Table 3. Univariate/PQ-treatment-adjusted predictors for time to *P. vivax* and *P. falciparum* episodes.**

| **Variable** | ***P. vivax*** | | | ***P. falciparum*** | | |
| --- | --- | --- | --- | --- | --- | --- |
|  | **HR^1^** | **CI_95_** | ***p*-value** | **HR^1^** | **CI_95_** | ***p*-value** |
| PQ treatment | 0.50 | 0.21-1.56 | 0.11 | 1.72 | 1.01-2.93 | 0.04 |
| *P. vivax* _mol_FOB^3^ | 1.07 | 1.05-1.09 | <0.001 | n.a. | n.a. | n.a. |
| *P. falciparum* _mol_FOB^3^ | n.a. | n.a. | n.a. | 1.18 | 1.15-1.21 | <0.001 |
| Age | 0.66 | 0.50-0.87 | 0.003 | 0.95 | 0.80-1.11 | 0.50 |
| LLIN at enrolment | 0.88 | 0.21-3.60 | 0.86 | 0.45 | 0.22-0.96 | 0.038 |
| Hb at enrolment (g/dl) | 0.76 | 0.58-0.99 | 0.045 | 0.76 | 0.63-0.93 | 0.006 |
| Village |  |  |  |  |  |  |
| Albinama (ref) | - | - | - | 1 | - | - |
| Amahup | 0.72 | 0.18-2.78 | 0.637 | 0.54 | 0.15-1.94 | 0.346 |
| Balif | 1.50 | 0.45-5.02 | 0.506 | 0.96 | 0.29-3.19 | 0.949 |
| Balanga | 0.68 | 0.16-3.02 | 0.617 | 1.25 | 0.47-3.36 | 0.656 |
| Bolumita | 2.12 | 0.69-6.53 | 0.189 | 3.78 | 1.61-8.90 | 0.002 |
| Numangu | 1.13 | 0.23-5.69 | 0.876 | 5.70 | 2.43-13.30 | 0.001 |
| Infection status at enrolment (by qPCR) | | | | | | |
| Uninfected (ref) | - | - | - | - | - | - |
| *P. vivax* | 2.41 | 0.94-6.18 | 0.068 | 3.21 | 1.67-6.16 | <0.001 |
| *P. falciparum* | 0.88 | 0.33-2.36 | 0.806 | 1.41 | 0.72-2.78 | 0.319 |
| Mixed *P.f.* or *P.v.* | 1.38 | 0.39-4.95 | 0.620 | 1.43 | 0.48-4.27 | 0.517 |

^1^ PQ treatment, univariate HR. All other HRs are adjusted only for PQ treatment. AHRs were modeled using multiple failure Cox proportional hazard regression.

^2^ n.a., not applicable

^3^ Average _mol_FOB until the time of failure (time-varying covariate).

**Table 4. Univariate/PQ-treatment-adjusted predictors for odds of *P. falciparum* clinical episodes.**

|  | ***P. falciparum* episode** | | |
| --- | --- | --- | --- |
| **Variable** | **OR^1^** | **CI_95_** | ***p-*value** |
| PQ treatment | 1.63 | 0.94-2.83 | 0.08 |
| *P. vivax* qPCR positive^2^ | 0.70 | 0.34-1.45 | 0.35 |
| *P. falciparum* _mol_FOB | 1.22 | 1.14-1.31 | <0.001 |
| Age | 0.89 | 0.76-1.06 | 0.20 |
| LLIN at enrolment | 0.39 | 0.18-0.86 | 0.02 |
| Haemoglobin (g/dl) | 0.97 | 0.85-1.11 | 0.68 |
| Village |  |  |  |
| Albinama (ref) | - | - |  |
| Amahup | 0.43 | 0.12-1.55 | 0.198 |
| Balif | 0.94 | 0.27-3.31 | 0.925 |
| Balanga | 1.34 | 0.46-3.85 | 0.592 |
| Bolumita | 4.14 | 1.59-10.74 | 0.004 |
| Numangu | 5.90 | 2.34-14.89 | <0.001 |
| Study Day | | | |
| Day 0-80 (ref) | - | - |  |
| Day 81-175 | 0.87 | 0.47-1.61 | 0.649 |
| Day >175 | 0.73 | 0.36-1.47 | 0.376 |

^1^ PQ treatment, univariate OR. All other ORs are adjusted only for PQ treatment. ORs were modeled using a binomial generalized estimating equation with logit link function using an exchangeable correlation structure.

^2^ Determined as *P. vivax* positive at the same or previous sampling visit.
