# Supplementary material for: The complex relationship of exposure to new Plasmodium infections and incidence of clinical malaria in Papua New Guinea
Source: eLife. 2017 Sep 1;6:e23708. doi: 10.7554/eLife.23708 (PMC5606846; doi:10.7554/eLife.23708)
Supplement: Supplementary file 2. [file elife-23708-supp2.docx]

**Supplementary File 2 - Multivariable predictors for time to recurrent blood-stage infection with *Plasmodium* species by LM.**

| **Variable** | ***P. vivax*** | | | ***P. falciparum*** | | |
| --- | --- | --- | --- | --- | --- | --- |
|  | **AHR^1^** | **CI_95_** | ***p*-value** | **AHR^1^** | **CI_95_** | ***p*-value** |
| PQ treatment | 0.17 | 0.11-0.24 | <0.001 | 0.76 | 0.50-1.17 | 0.216 |
| Age | 0.85 | 0.77-0.95 | 0.004 | 1.16 | 1.01-1.33 | 0.037 |
| LLIN use at enrolment | 0.76 | 0.45-1.30 | 0.319 | 0.56 | 0.31-1.02 | 0.056 |
| Hb at enrolment (g/dl) | 0.86 | 0.76-0.97 | 0.18 | 0.78 | 0.67-0.91 | 0.002 |
| Village |  |  |  |  |  |  |
| Albinama (ref) | 1 |  |  | 1 |  |  |
| Amahup | 0.35 | 0.19-0.63 | 0.001 | 1.19 | 0.44-3.20 | 0.736 |
| Balanga | 1.49 | 0.88-2.51 | 0.139 | 3.17 | 1.24-8.12 | 0.016 |
| Balif | 1.12 | 0.69-1.84 | 0.644 | 1.25 | 0.45-3.46 | 0.673 |
| Bolumita | 3.38 | 1.97-5.82 | <0.001 | 9.07 | 3.76-21.87 | <0.001 |
| Numangu | 0.59 | 0.25-1.35 | 0.208 | 5.93 | 2.27-15.50 | <0.001 |
| Infection status at enrolment (by qPCR) | | | | | |  |
| Uninfected (ref) | 1 |  |  | 1 |  |  |
| *P. vivax* | 1.49 | 0.98-2.25 | 0.062 | 1.21 | 0.63-2.35 | 0.562 |
| *P. falciparum* | 1.10 | 0.61-1.99 | 0.747 | 1.53 | 0.74-3.15 | 0.251 |
| *P. malariae* | 1.28 | 0.53-3.11 | 0.575 | 0.87 | 0.25-2.99 | 0.828 |
| Mixed *P.f. or P.v*.^2^ | 1.94 | 1.18-3.21 | 0.009 | 2.24 | 1.18-4.23 | 0.013 |

^1^ AHRs were modeled using Cox proportional hazard regression.

^2^ Mixed infection including *P. falciparum* or *P. vivax* infection in conjunction with one or more other *Plasmodium spp.*
