# Supplementary material for: The complex relationship of exposure to new Plasmodium infections and incidence of clinical malaria in Papua New Guinea
Source: eLife. 2017 Sep 1;6:e23708. doi: 10.7554/eLife.23708 (PMC5606846; doi:10.7554/eLife.23708)
Supplement: Supplementary file 3. [file elife-23708-supp3.docx]

**Supplementary file 3 - Multivariable predictors for *P. falciparum* and *P. vivax* density by qPCR during follow-up.**

|  | ***P. vivax* density^1^ by qPCR** | | | ***P. falciparum* density^1^ by qPCR** | | |
| --- | --- | --- | --- | --- | --- | --- |
| Variable | **exp(β)^2^** | **CI_95_** | ***p*-value** | **exp(β)^2^** | **CI_95_** | ***p*-value** |
| PQ treatment | 1.16 | 0.85-1.58 | 0.354 | 0.66 | 0.39-1.10 | 0.112 |
| Fever (>37.5°C axillary) | 0.99 | 0.57-1.71 | 0.960 | 7.38 | 3.60-15.14 | <0.001 |
| *P. falc.* _mol_FOB | n.a. | n.a. | n.a. | 1.07 | 1.00-1.15 | 0.039 |
| P. *vivax* qPCR positive | n.a. | n.a. | n.a. | 0.45 | 0.25-0.81 | 0.008 |
| *P. vivax* _mol_FOB | 1.03 | 1.01-1.05 | 0.006 | n.a. | n.a. | n.a. |
| P. *falc.* qPCR positive | 1.11 | 0.81-1.52 | 0.529 | n.a. | n.a. | n.a. |
| Age | 0.90 | 0.83-0.98 | 0.016 | 0.96 | 0.80-1.14 | 0.645 |
| LLIN at enrol. | 1.03 | 0.71-1.49 | 0.883 | 0.41 | 0.15-1.08 | 0.072 |
| Hb at enrolment (g/dl) | 1.11 | 1.00-1.22 | 0.050 | 0.91 | 0.74-1.11 | 0.353 |
| Village |  |  |  |  |  |  |
| Albinama | 1 |  |  | 1 |  |  |
| Amahup | 1.02 | 0.56-1.86 | 0.941 | 0.83 | 0.21-3.36 | 0.796 |
| Balif | 0.99 | 0.71-1.37 | 0.933 | 1.26 | 0.38-4.14 | 0.707 |
| Balanga | 0.71 | 0.49-1.03 | 0.071 | 3.35 | 0.78-14.43 | 0.104 |
| Bolumita | 0.79 | 0.57-1.10 | 0.168 | 0.76 | 0.26-2.27 | 0.629 |
| Numangu | 0.93 | 0.48-1.81 | 0.833 | 0.82 | 0.23-2.91 | 0.757 |
| Month of follow-up | 0.77 | 0.73-0.82 | <0.001 | 0.82 | 0.71-0.94 | 0.004 |

^1^ Parasite densities by qPCR were assessed as *18S rRNA* copy numbers/µl blood and log_10_ transformed.

^2^ Estimates were obtained using gaussian generalized estimating equations with logit-link allowing for repeated visits.

^3^ n.a., not applicable
