# Supplementary material for: The complex relationship of exposure to new Plasmodium infections and incidence of clinical malaria in Papua New Guinea
Source: eLife. 2017 Sep 1;6:e23708. doi: 10.7554/eLife.23708 (PMC5606846; doi:10.7554/eLife.23708)
Supplement: Supplementary file 4. — This model is similar to that presented in Table 4 in the main text but combines the treatment arms for P. vivax. Model predictions from this model were used for mapping molFOI in Figure 3B. [file elife-23708-supp4.docx]

**Supplementary file 4 - Multivariable predictors of *Pv-*_mol_FOB (combining primaquine and placebo arms) per follow-up interval.** This model is similar to that presented in Table 4 in the main text but combines the treatment arms for *P. vivax*. Model predictions from this model were used for mapping molFOI in Figure 3B.

| **Variable** | ***P. vivax (combined)*** | | |
| --- | --- | --- | --- |
|  | **IRR^1^** | **CI_95_** | ***p*-value** |
| PQ treatment | 0.27 | 0.2-0.36 | <0.001 |
| New *P. falc.* infections in interval^2^ | 1.18 | 0.96-1.45 | 0.113 |
| Age | 0.92 | 0.85-1 | 0.043 |
| LLIN at enrolment | 0.74 | 0.54-1.01 | 0.057 |
| Hb at enrolment (g/dL) | 0.9 | 0.84-0.97 | 0.007 |
| Village |  |  |  |
| Albinama (ref) | 1 |  |  |
| Amahup | 0.34 | 0.21-0.56 | <0.001 |
| Balif | 1.39 | 0.95-2.03 | 0.087 |
| Balanga | 0.78 | 0.51-1.18 | 0.233 |
| Bolumita | 2.22 | 1.52-3.23 | <0.001 |
| Numangu | 0.64 | 0.35-1.16 | 0.138 |
| Study Day |  |  |  |
| Day 0-35 (ref) | 1 |  |  |
| Day 36-80 | 1.81 | 1.28-2.57 | 0.001 |
| Day 81-175 | 1.01 | 0.71-1.44 | 0.945 |
| Day >175 | 0.58 | 0.4-0.85 | 0.005 |

^1^IRRs were modeled per sampling interval using negative binomial generalized estimating equations allowing for repeated visits with log-link and an exchangeable correlation structure.

^2^ _mol_FOB in the follow-up interval (time-varying covariate).
