# Supplementary material for: The complex relationship of exposure to new Plasmodium infections and incidence of clinical malaria in Papua New Guinea
Source: eLife. 2017 Sep 1;6:e23708. doi: 10.7554/eLife.23708 (PMC5606846; doi:10.7554/eLife.23708)
Supplement: Transparent reporting form [file elife-23708-transrepform.docx]

***eLife’s* transparent reporting form**

We encourage authors to provide detailed information *within their submission* to facilitate the interpretation and replication of experiments. If you have any questions, please contact us: [editorial@elifesciences.org](mailto:editorial@elifesciences.org).

**Sample-size estimation**

- You should state whether an appropriate sample size was computed when the study was being designed
- You should state the statistical method of sample size computation and any required assumptions
- If no explicit power analysis was used, you should describe how you decided what sample (replicate) size (number) to use

Please outline where this information can be found within the submission (e.g., page numbers or figure legends), or explain why this information doesn’t apply to your submission:

Detailed sample size computations can be found in a previous manuscript that describes the cohort study that this dataset is derived from in a high level of detail. This manuscript is cited in the methods section on page 25:

Reference 23 - Robinson LJ, et al. Strategies for Understanding and Reducing the *Plasmodium vivax* and *Plasmodium ovale* Hypnozoite Reservoir in Papua New Guinean Children: A Randomised Placebo-Controlled Trial and Mathematical Model. PLoS Med. 2015 Oct;12(10):e1001891.

**Replicates**

- You should report how often each experiment was performed
- You should include a definition of biological versus technical replication
- The data obtained should be provided and sufficient information should be provided to indicate the number of independent biological and/or technical replicates
- If you encountered any outliers, you should describe how these were handled
- Criteria for exclusion/inclusion of data should be clearly stated
- High-throughput sequence data should be uploaded before submission, with a private link for reviewers provided (these are available from both GEO and ArrayExpress)

Please outline where this information can be found within the submission (e.g., page numbers or figure legends), or explain why this information doesn’t apply to your submission:

Sections 4.1 (Methods – Study design and participants; Page 25) and 2.1 (Results – parasitological parameters at enrolment; Page 5) describe inclusion/exclusion criteria for enrolment in the study and data analysis, respectively. Detailed description of exclusion/inclusion criteria can also be found in reference 23, which is cited in the methods section on page 25.

Section 4.2 (Methods – laboratory methods; Page 26) describes the workflow of laboratory procedures. Due to the large number of samples in this longitudinal population-based study, biological tests such as diagnostic or genotyping PCR were performed in simplicate for each sample at the Swiss Tropical and Public Health Institute (Swiss TPH). Rigorous external and internal quality control procedures for qPCR and genotyping PCR are in place at SwissTPH and include exchange of specimens with partner institutions and participation in international quality control schemes e.g. UK NEQAS.

**Statistical reporting**

- Statistical analysis methods should be described and justified
- Raw data should be presented in figures whenever informative to do so (typically when N per group is less than 10)
- For each experiment, you should identify the statistical tests used, exact values of N, definitions of center, methods of multiple test correction, and dispersion and precision measures (e.g., mean, median, SD, SEM, confidence intervals; and, for the major substantive results, a measure of effect size (e.g., Pearson's r, Cohen's d)
- Report exact p-values wherever possible alongside the summary statistics and 95% confidence intervals. These should be reported for all key questions and not only when the p-value is less than 0.05.

Detailed descriptions of statistical analysis, including structure and parameters of models applied to analyze raw data, can be found in section 4.3 (Methods – statistical analysis; Page 27). A detailed description of each statistical test or model, as well as detailed description of variables tested and excluded to obtain most parsimonious model, can be found as footnote to each result table.

A graphical presentation of the method using to calculate of molFOB, the main parameter in this manuscript, can be found in supplementary Figure 1 – figure supplement 1 (Page 32-33). Distribution of raw molFOB values in the study population is presented in Figure 2 (Page 13).

Please outline where this information can be found within the submission (e.g., page numbers or figure legends), or explain why this information doesn’t apply to your submission:

(For large datasets, or papers with a very large number of statistical tests, you may upload a single table file with tests, Ns, etc., with reference to page numbers in the manuscript.)

**Additional data files (“source data”)**

- We encourage you to upload relevant additional data files, such as numerical data that are represented as a graph in a figure, or as a summary table
- Where provided, these should be in the most useful format, and they can be uploaded as “Source data” files linked to a main figure or table
- Include model definition files including the full list of parameters used
- Include code used for data analysis (e.g., R, MatLab)
- Avoid stating that data files are “available upon request”

Please indicate the figures or tables for which source data files have been provided:

No source data for graphs or tables are provided with this submission, as the majority of data presented in figures or graphs are also stated as numerical data within the text in the results section, or presented as raw data such as in Figure 2 (Page 13).
